# Reactions of Arsenoplatin-1 with Protein Targets: A Combined Experimental and Theoretical Study

**DOI:** 10.1021/acs.inorgchem.1c03732

**Published:** 2022-02-09

**Authors:** Iogann Tolbatov, Damiano Cirri, Matteo Tarchi, Tiziano Marzo, Cecilia Coletti, Alessandro Marrone, Luigi Messori, Nazzareno Re, Lara Massai

**Affiliations:** † Institut de Chimie Moleculaire de l’Université de Bourgogne (ICMUB), Université de Bourgogne Franche-Comté (UBFC), Avenue Alain Savary 9, 21078 Dijon, France; ‡ Department of Chemistry and Industrial Chemistry, University of Pisa, Via G. Moruzzi 13, 56124 Pisa, Italy; § Department of Chemistry, University of Florence, Via della Lastruccia 3-13, 50019 Sesto Fiorentino, Italy; ∥ Department of Pharmacy, University of Pisa, Via Bonanno Pisano 6, 56126 Pisa, Italy; ⊥ CISUP - Centre for Instrumentation Sharing (Centro per l’Integrazione della Strumentazione Scientifica), University of Pisa, 56126 Pisa, Italy; # University Consortium for Research in the Chemistry of Metal ions in Biological Systems (CIRCMSB), Via Celso Ulpiani 27, 70126 Bari, Italy; ∇ Dipartimento di Farmacia, Università “G d’Annunzio” di Chieti-Pescara, Via dei Vestini 31, 66013, Chieti, Italy

## Abstract

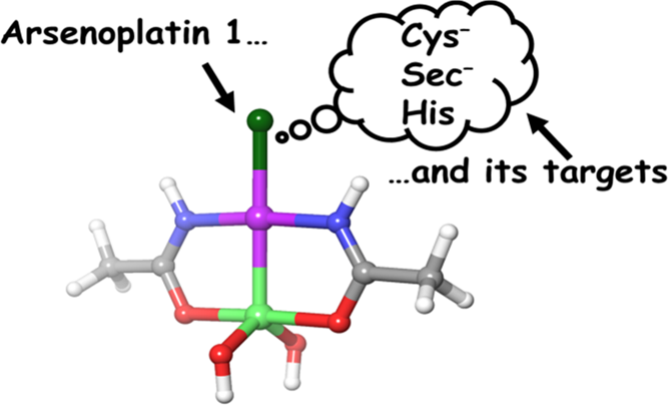

Arsenoplatin-1 (AP-1) is a dual-action anticancer metallodrug with a promising pharmacological profile that features the simultaneous presence of a cisplatin-like center and an arsenite center. We investigated its interactions with proteins through a joint experimental and theoretical approach. The reactivity of AP-1 with a variety of proteins, including carbonic anhydrase (CA), superoxide dismutase (SOD), myoglobin (Mb), glyceraldehyde 3-phosphate dehydrogenase (GAPDH), and human serum albumin (HSA), was analyzed by means of electrospray ionization mass spectrometry (ESI MS) measurements. In accordance with previous observations, ESI MS experiments revealed that the obtained metallodrug–protein adducts originated from the binding of the [(AP-1)-Cl]^+^ fragment to accessible protein residues. Remarkably, in two cases, i.e., Mb and GAPDH, the formation of a bound metallic fragment that lacked the arsenic center was highlighted. The reactions of AP-1 with various nucleophiles side chains of neutral histidine, methionine, cysteine, and selenocysteine, in neutral form as well as cysteine and selenocysteine in anionic form, were subsequently analyzed through a computational approach. We found that the aquation of AP-1 is energetically disfavored, with a reaction free energy of +19.2 kcal/mol demonstrating that AP-1 presumably attacks its biological targets through the exchange of the chloride ligand. The theoretical analysis of thermodynamics and kinetics for the ligand-exchange processes of AP-1 with His, Met, Cys, Sec, Cys^–^, and Sec^–^ side chain models unveils that only neutral histidine and deprotonated cysteine and selenocysteine are able to effectively replace the chloride ligand in AP-1.

## Abstract 2

The interaction of arsenoplatin-1 (AP-1), a promising dual-action anticancer metallodrug featuring the simultaneous presence of a cisplatin-like center and an arsenite center, with proteins was studied via a synergetic experimental and theoretical approach. The reactivity of AP-1 with a variety of proteins was analyzed through ESI MS measurements and, at the same time, the reactions of AP-1 with molecular models of the side chains of histidine, methionine, cysteine, and selenocysteine were analyzed through a computational approach.

## 1 Introduction

Transition-metal complexes are widely used in medicinal chemistry;^1−4^ the case of cisplatin as an anticancer agent being the most representative.^5^ As a matter of fact, there is a continuous interest in the development of cisplatin derivatives with the objective of ameliorating their antitumor potency while decreasing systemic toxicity.^6−8^ The toxicity of cisplatin originates from the relatively easy *in vivo* replacement of the chloride ligands by donor atoms of endogenous targets; actually, the testing of a plethora of less reactive Pt ligands in the place of chloride has permitted the production of metallodrugs with lower systemic toxicity and a higher therapeutic index.^9−13^ This proves that the design of new active Pt(II)-based compounds should involve the structure-based control of the substitution reaction.^14,15^

**Figure 1:**
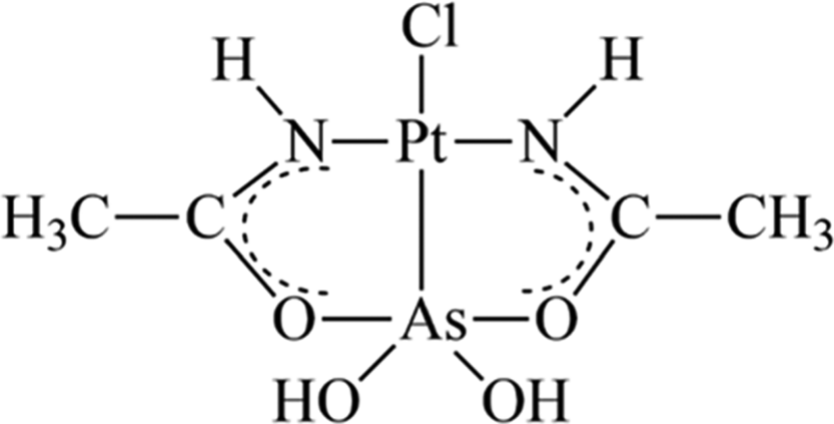
Chemical structure of arsenoplatin-1.

Arsenoplatin-1 (AP-1) is a novel dual-action metallodrug characterized by an antitumor effect based on the synergetic interplay of a square planar Pt(II) center and the coordinated arsenic trioxide moiety^16^ (Figure 1), resulting in a superior antitumor activity in a majority of cancer cell lines.^16,17^

The mechanism of action of AP-1 is not yet completely comprehended at the molecular level, although several experimental^16−19^ and computational^20−22^ investigations have been reported so far. AP-1 binding to DNA was studied by inductively coupled plasma mass spectrometry on AP-1-DNA adducts extracted from triple-negative breast MDA-MB-231 cancer cells, revealing that gradual and continuous release of the As(OH)_2_ moiety inside the cell results in the augmented toxicity of arsenoplatin-1 juxtaposed to cisplatin.^16^ Density-functional theory (DFT) calculations unveiled that guanine is a more favored binding site than adenine for AP-1 as well as for other platinum-containing compounds.^20^ It was also shown that the hydrolysis of AP-1 necessitates a higher energy barrier than that for DNA platination, although the barrier for aquation is lower than that of cisplatin because of the trans effect of the arsenic moiety.^20^ A detailed computational study of the metalation of the bovine pancreatic ribonuclease (RNase A) by AP-1 revealed the binding of His to platinum(II), retaining the Pt–As bond. The computations evidenced that the metalation is more advantageous in water than in the protein milieu, consistent with the character of the protein binding pocket residues.^22^

Interestingly, the replacement of chloride in AP-1 with iodide did not hamper its cytotoxicity, thus proving that the Pt–As core is the “true” cytotoxic metal scaffold.^23^

Given the differences in the mechanism of action of arsenoplatin compared to cisplatin, it is plausible to assume that the interactions with proteins may play a prominent part in the action mode of AP-1. To the best of our knowledge, there are only two investigations of the reactivity of AP-1 with proteins. The first study focuses on the interactions of AP-1 with the small model proteins hen egg-white lysozyme (HEWL) and bovine pancreatic ribonuclease (RNase A). The corresponding crystal structures of AP-1-protein adducts revealed that the preferred binding sites for AP-1 are the His side chains in both proteins.^16^ Unlike cisplatin and carboplatin, which target the sulfurs of Met side chains of RNase A,^24^ AP-1 does not show any preference for Met side chains. Another evidence of AP-1 targeting His residue was offered by a recent study, in which AP-1 was placed into the apoferritin (AFt) nanocage; the resulting X-ray structure revealed the coordination of the AP-1 fragment to the side chain of a His residue.^19^

The present study has a twofold objective.

On one hand, we aim to expand the knowledge of the reactions of AP-1 with proteins by considering a larger and more representative group of proteins including human carbonic anhydrase 1 (*h*CA1), bovine superoxide dismutase (SOD), horse heart myoglobin (Mb), glyceraldehyde 3-phosphate dehydrogenase (GAPDH) from rabbit muscle, and human serum albumin (HSA). These reactions and the associated adduct formation were examined through the classical ESI MS strategy developed in our laboratory.

**Scheme 1:**
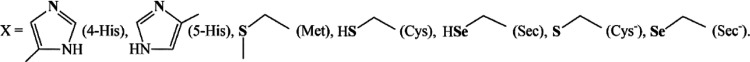
Simplified Models for Protein Residues Employed in DFT Calculations

On the other hand, the reactions of the neutral AP-1 with water and the histidine, methionine, cysteine, and selenocysteine side chains, which are the main candidates for protein metalation by AP-1, are investigated by means of DFT approaches to shed light on the observed binding preferences of protein residues for AP-1. More specifically, we have employed simple (short) models of these residues where each side chain is modeled by the nucleophilic group, *i.e.*, imidazole, CH_3_S^–^, HS^–^, and HSe^–^ for His, Met, Cys, and Sec residues, respectively, whereas the remainder of the chain is rendered by an ethyl group (Scheme 1).

**Scheme 2:**
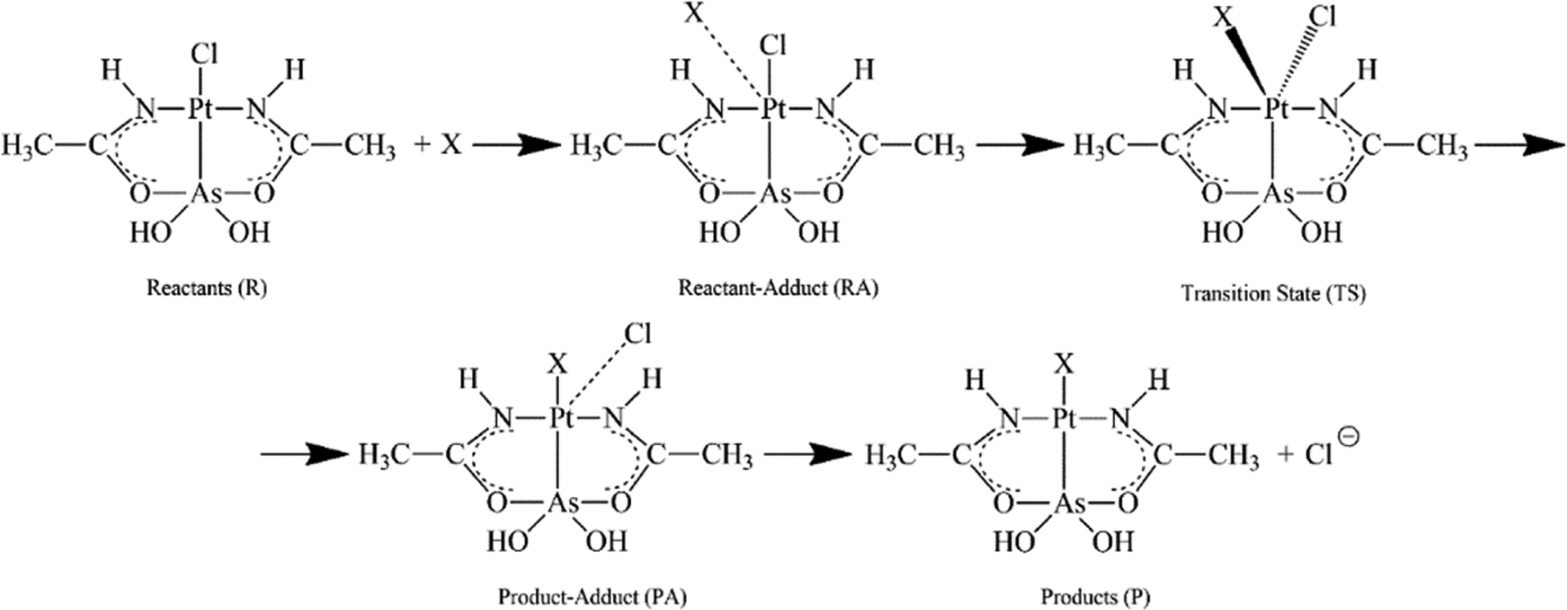
Reaction Scheme for the Nucleophile Substitution on the AP Complex Explicitly Showing the Intermediate and Transition State Species

Moreover, we assume that the reaction of AP-1 with protein targets occurs through the nucleophilic substitution on the Pt(II) center of the labile chloride ligand by the entering X ligand via an associative interchange mechanism as depicted in Scheme 2.

To gain a broader comprehension of the binding mechanism of AP-1 with protein targets as well as of their binding preference, we have evaluated both the thermodynamics and kinetics of the proposed mechanistic hypotheses. Indeed, computational studies were frequently and auspiciously employed for the characterization of the reactivity of metals and metallodrugs with proteins.^25−28^ Understanding the binding preference of AP-1 would be very beneficial to completely understand its mechanism of action *in vivo* and may be advantageous to design more efficacious anticancer drugs.

## 2 Results

### 2.1 Reactions of AP-1 with a Few Representative Proteins Analyzed by ESI MS

The reactions of AP-1 with the model proteins HEWL and RNase A were studied by ESI MS measurements in our previous study.^16^ Those results clearly showed that AP-1 binds both proteins through coordination of an [(AP-1)-Cl]^+^ fragment after the release of the Cl^–^ ligand. Complementary X-ray diffraction studies revealed that this metallic fragment was coordinated at the level of His15 of HEWL and His105 and His119 residues in the case of RNase A.

Here, we have extended this type of approach to a larger number of proteins some of them also being of a considerably greater size. Specifically, the following proteins were employed for this new study: *h*CA1, SOD, Mb, GAPDH, and HSA.

The interactions of these proteins with AP-1 were investigated according to a standard experimental setup including preparation of the protein solution in 2 × 10^–3^ M ammonium acetate at pH 6.8; addition of a threefold excess of AP-1; incubation delay; recording of the ESI MS spectra.

**Figure 2:**
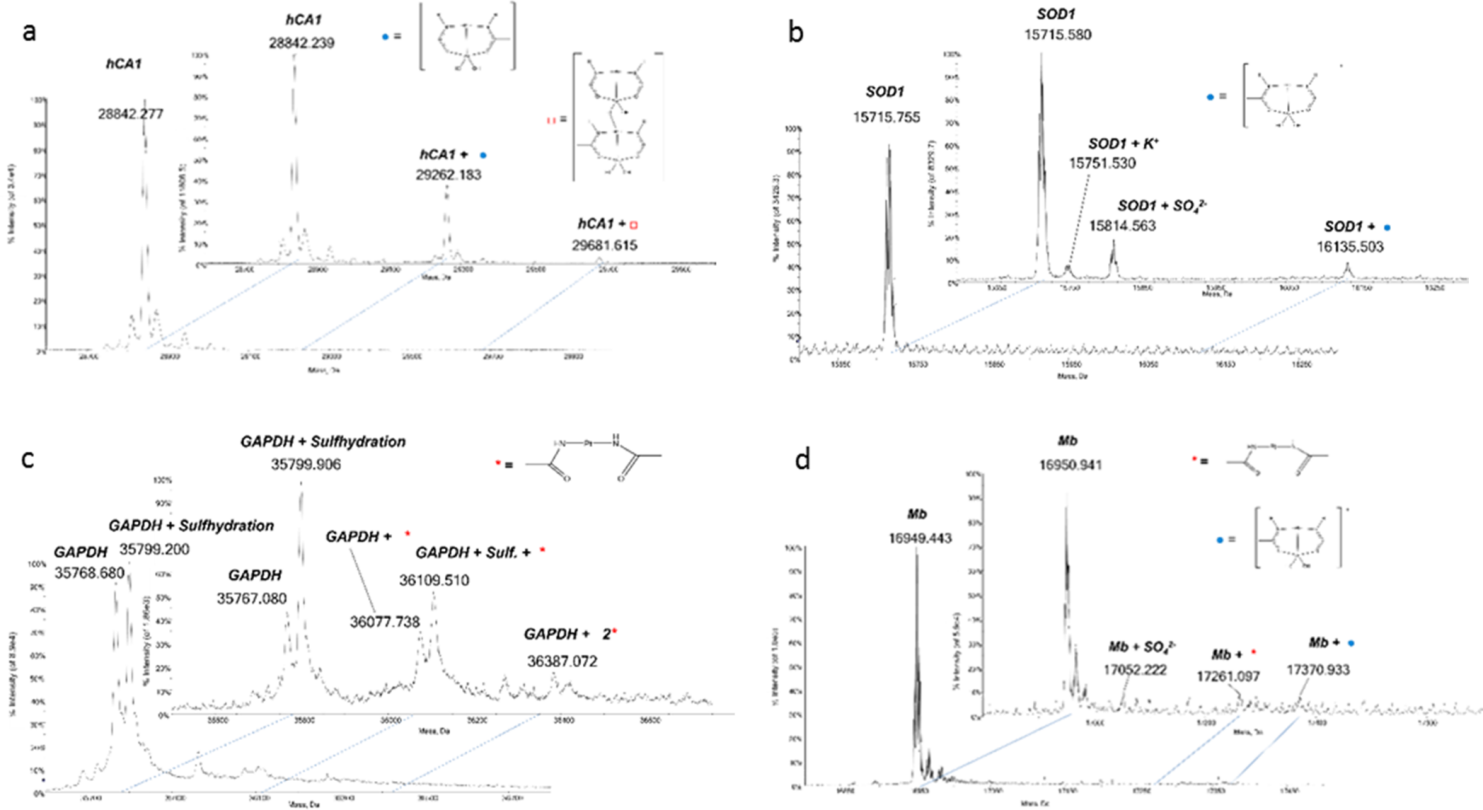
(a) Deconvoluted mass spectrum of human carbonic anhydrase 1 (hCA1) overlapped to the deconvoluted mass spectrum of AP-1 incubated with *h*CA1, at 37 °C for 3 h in 1:3 protein-to-AP-1 ratio. (b) Deconvoluted mass spectrum of superoxide dismutase 1 (SOD1) overlapped with the deconvoluted mass spectrum of AP-1 incubated with SOD1, at 37 °C for 3 h in 1:3 protein-to-AP-1 ratio. (c) Deconvoluted mass spectrum of glyceraldehyde 3-phosphate dehydrogenase (GAPDH) overlapped to the deconvoluted mass spectrum of AP-1 incubated with GAPDH, at 37 °C for 3 h in 1:3 protein-to-AP-1 ratio, with 0.1% v/v of formic acid shortly prior to the infusion in the mass spectrometer. (d) Deconvoluted mass spectrum of myoglobin (Mb) overlapped with the deconvoluted mass spectrum of AP-1 incubated with Mb, at 37 °C for 3 h in 1:3 protein-to-AP-1 ratio, with 0.1% v/v of formic acid shortly prior to the infusion in the mass spectrometer.

The resulting deconvoluted ESI MS spectra are reported in Figure 2, with a direct comparison of the proteins’ spectra after and before the AP-1 addition. Particularly, the metalation of all tested proteins already took place after 3 h of AP-1 incubation.

The interpretation of these ESI MS spectra is quite straightforward. In most cases, AP-1-protein adducts are formed as witnessed by the appearance of new peaks of a greater mass; however, the amount of the formed adducts is quite limited. In most cases, a peak characterized by a mass shift of +419 is detected. This mass increase well matches the mass of an [(AP-1)-Cl] fragment, or of its dimer (in the case of *h*CA1), in line with previous observations.^29^

The spectra of the tested proteins, after 24 h incubation, typically show a net decrease in the intensity of the adducts signal, suggesting a progressive instability of the binding.

The spectrum of the GAPDH protein is characterized by two major signals; one, at 35 764 Da, assigned to the native protein, and another, at 35 797 Da, probably due to the Cys150 sulfhydration; this double signal is also detected in the spectrum of the AP-1 adducts.

Interestingly, in the case of both GAPDH and Mb, a new fragment of a different mass, with a shift of 311 Da, is observed. Notably, this mass shift well corresponds to the Pt(NHC(CH_3_)O)_2_ fragment. Observation of this fragment offers direct evidence that arsenoplatin-1, upon interaction with certain proteins, may undergo the breaking of the As–Pt bond and the detachment of the As(OH)_2_ group, an interesting feature that had not been observed in the previous studies.

In addition, AP-1 has been tested with HSA, the main plasma protein; we found that AP-1 manifests the tendency to react again, forming an adduct with the Pt(NHC(CH_3_)O)_2_ fragment. In Figure S1, Supporting Information, the mass spectra of HSA before and after the addition of AP-1 are presented. The deconvoluted mass spectrum of metal-free HSA is marked by the signals at 66 438 and 66 557 Da, corresponding to the protein in its native and its cysteinylated forms, respectively, *i.e.*, the protein with a Cys residue bound to the Cys34. Interestingly, AP-1 upon reacting with HSA produces adducts with both the native and the cysteinylated proteins, primarily with the native protein as evidenced by the lower intensity of the native protein signal with respect to the cysteinylated protein signal.

### 2.2 Computational Studies

**Figure 3:**
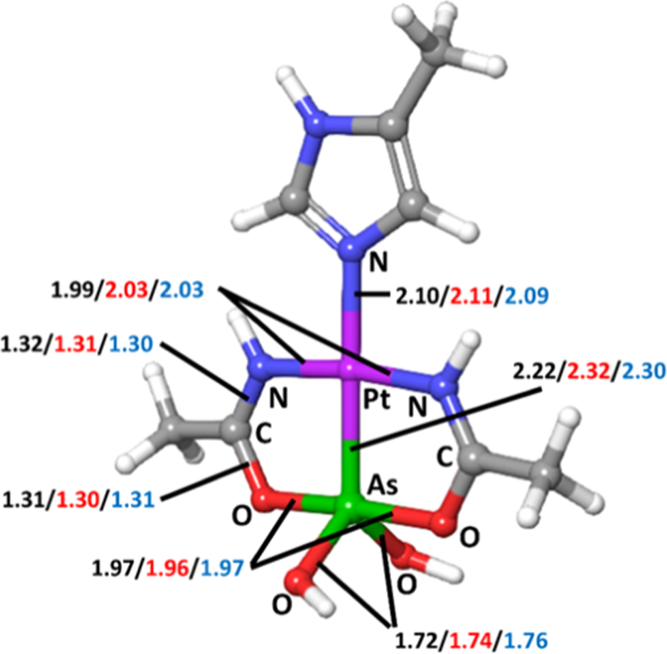
Representation of the experimentally characterized structure of AP-1-His (4-His) complex.^16^ The reported values of bond distances are experimentally measured (black), optimized in the gas phase (red), and optimized in the solvated phase (blue). All distances are in angstroms.

A preliminary investigation on the AP-SCN and AP-1 complexes^21^ has shown that the range-corrected CAM-B3LYP density functional with the LANL2DZ effective core potential and the 6-31+G* basis set yields the minimized structures well matching the crystallographic data^17^ with Pt–S and Pt–As bond distances within 0.04 Å error. We extracted the AP-1-His complex from the X-ray crystallographic data for the AP-1-HEWL adduct^16^ and made a comparison of geometrical parameters obtained by experiment, optimization in the gas phase, and optimization in water (Figure 3). We can see that most of the calculated bond distances, including the crucial metal center–ligand bond Pt–N, were estimated within an error of 0.01 Å, whereas the calculated Pt–As bond is within 0.1 and 0.08 Å for the optimization in the gas phase and solvated phase, respectively.

The thermodynamics and kinetics of the ligand substitution of chloride by entering nucleophile molecules were analyzed via DFT computations. The choice of modeling the investigated protein residues with the simplified models shown in Scheme 1, instead of capped or free amino acids, requires some further consideration.^30^ On one hand, the capped forms of amino acids are linked to the nucleophilic groups of side chains via hydrocarbon chains of different lengths, which leads to considerable variation in the size of the ligand interacting with metal complex and thus substantially affecting the computation of solvation free energies. On the other hand, free amino acids contain terminus carboxylic acid and amine groups in ionized zwitterionic form, which do not exist in proteins. We assume all residues to exist in their most stable protonation state at pH = 7.2: histidine and methionine are neutral, whereas selenocysteine is anionic (however, we included the neutral form of selenocysteine for completeness). Both neutral and anionic forms of cysteine were considered since both are present at neutral pH, although the anionic form can be found only in low concentrations. Moreover, the anionic form of cysteine might be stabilized in the vicinity of histidine and other basic residues.

We assume that the ligand-exchange reactions on AP-1 undergo an associative interchange mechanism, with reactants and products forming stable noncovalent adducts before and after the reaction. Thus, the geometries of the reactants (R), reactant adducts (RA), transition states (TS), product adducts (PA), and products (P) were calculated. The activation enthalpies and free energies were calculated as the difference between TS and the lowest between reactants and reactant adducts, whereas the reaction enthalpies and free energies were calculated as the difference between reagents and products infinitely apart.

Initially, we analyzed the hydrolysis reaction with the exchange of chloride by a water molecule to test the stability of AP-1 in biological fluids and consequently determine if the chlorido or the aquo form of AP-1 might be the reactive species with protein targets. Indeed, it is well known that cisplatin and other biologically active metal complexes^31−33^ often go through hydrolytic activation, at which the labile aquo ligand replaces at least one ligand of the metal center. The calculated reaction free energy value for the aquation of AP-1 is 19.2 kcal/mol, a similar value to that calculated in ref (20). This value shows that this reaction is thermodynamically unfavorable and is improbable to take place at physiological temperature, indicating that AP-1 is expected to attack the biomolecular targets with the metal center in its chlorido form.

**Figure 4:**
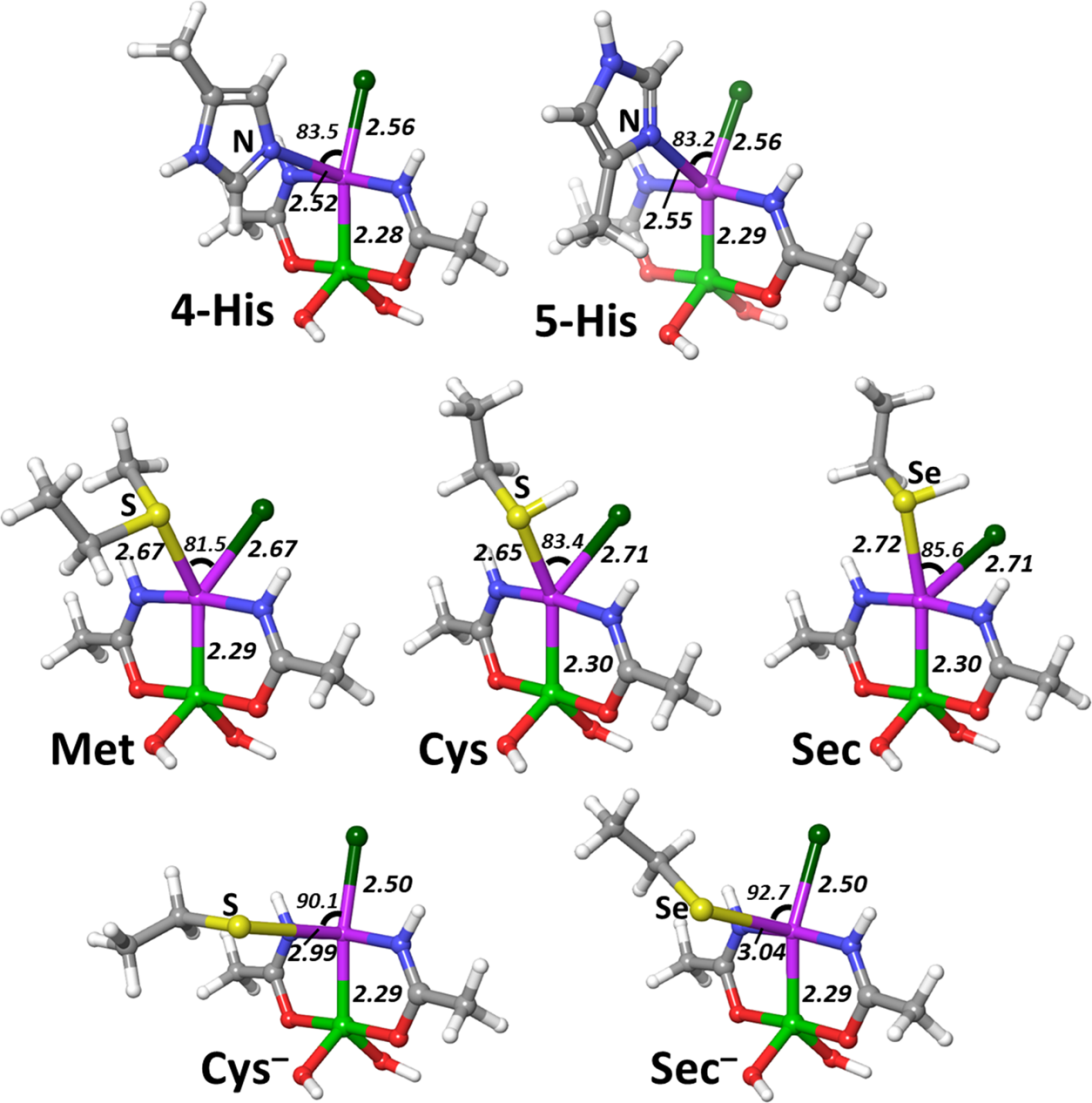
Transition states for the considered nucleophiles. All distances are in angstroms and all angles in degrees.

The reaction of AP-1 with models of the His, Met, Cys, and Sec side chains was consequently investigated assuming the associative interchange mechanism in each case. The optimized geometries of transition state structures allow us to obtain interesting insights into the reactivity disclosed by the analyzed protein residues. For example, the Pt–As bond in all calculated transition states is 2.28–2.30 Å, suggesting that the trans effect is not observed in the geometrical structure with a trigonal bipyramidal or square pyramidal configuration. The transition state geometries for the reaction of AP-1 with the neutral side chain models are characterized by an approximately trigonal bipyramidal coordination of the Pt center (Figure 4) with all entering ligand–Pt-leaving Cl angles ranging from 81.5 to 85.6°. The Pt–N distances of 2.52 and 2.55 Å are observed for 4-His and 5-His, respectively, and the same Pt–Cl distance of 2.56 Å, which suggests that both tautomers disclose almost the same reactivity. The Pt–S/Se bond lengths of 2.67–2.72 Å correspond well with the 2.67–2.71 Å lengths of Pt–Cl bonds for the transition states of neutral Cys, Sec, and Met, indicating that these transition states are neither early nor late. Deprotonated cysteine and selenocysteine, the only anionic nucleophiles in this study, form somewhat distorted square pyramidal transition states with S–Pt–Cl and Se–Pt–Cl angles of 90.1 and 92.7° for Cys^–^ and Sec^–^, respectively. Pt–S/Se bonds are 2.99/3.04 Å, while the Pt–N distances are 2.50 Å, this suggests very early transition states (Figure 4).

**Figure 5:**
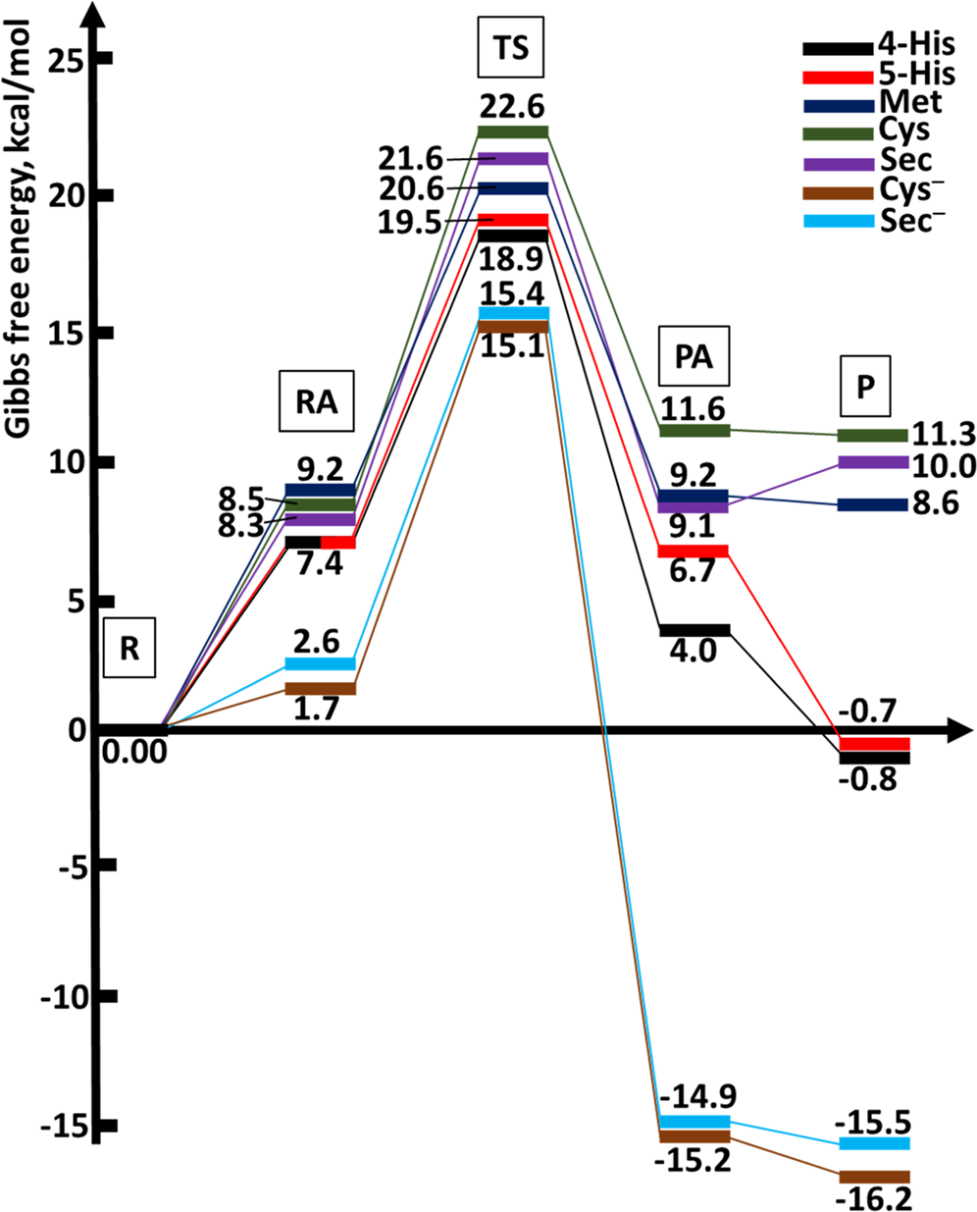
Reaction profiles for AP-1 reacting with the considered nucleophiles. Values in kcal/mol are computed in solution at the CAM-B3LYP/LANL08(f)/6-311++G**//CAM-B3LYP/LANL2DZ/6-31+G* level.

The computed values for the reaction enthalpy and free energy for the ligand substitution reactions involving the exchange of chloride with the investigated models (Table S1 and Figure 5) permit us to determine the thermodynamic preferences for Pt(II) binding to the examined side chains of protein residues. Calculations indicate that the reaction of AP-1 with the neutral Cys, Sec, and Met is moderately endothermic and endergonic, whereas the reaction with His and deprotonated Cys^–^ and Sec^–^ is exothermic and exergonic. It is also worth noticing that 5-His and 4-His have very close reaction and activation free energies. In accordance with the calculated thermodynamics, we are able to finally establish the regiochemistry for the reaction of AP-1 with the tested side chain models in the trend Cys^–^ > Sec^–^ > 5-His∼4-His > Met > Sec > Cys, hence noting the significance of the protonation state of the nucleophile.

The activation free energies for the reaction of AP-1 with 5-His and 4-His are 19.5 and 18.9 kcal/mol, respectively, resulting in the lowest barriers among the considered neutral residues. Moreover, the reaction free energies of −0.7 and −0.8 kcal/mol for 5-His and 4-His, respectively, make them the only neutral protein side chains targeted by AP in both exothermic and exergonic processes. The reaction of AP-1 with either Met, Cys, or Sec model is affected by a higher activation free energy, i.e., 20.6, 22.6, or 21.6 kcal/mol, respectively, as well as higher reaction free energies of 8.6, 11.3, and 10.0 kcal/mol in the same order, respectively. Our calculations clearly indicate the chloride substitution by His results to be both thermodynamically and kinetically most favorable compared to the other neutral side chain models.

On the other hand, the reaction of AP-1 with cysteine and selenocysteine in their anionic forms results to be both thermodynamically and kinetically the most favorable. Indeed, the computed activation free energy values for the exchange of chloride ligand with Cys^–^ and Sec^–^ are only 15.1 and 15.4 kcal/mol, respectively, whereas the corresponding values for the reaction free energy are −16.2 and −15.5 kcal/mol.

An overall insight into both thermodynamics and kinetics of the ligand substitution reactions investigated in the present study is provided in Figure 5. The results suggest that AP is expected to preferentially bind at His residues unless deprotonated cysteine or selenocysteine are available.

The binding preference in the AP-1 protein targeting obtained by our computational models implicitly assumes the same steric accessibility of these residues. However, protein side chains characterized by a high solvent exposure are more reachable to the AP complex and thus expected to be more reactive.

**Figure 6:**
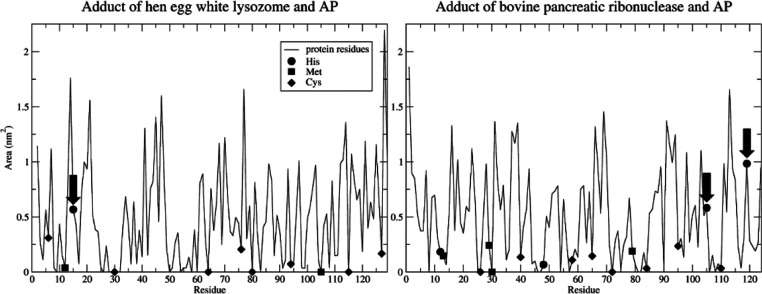
Solvent-accessible surface (SAS) computed on the X-ray structures of adducts of AP with two small model proteins, hen egg-white lysozyme (HEWL), and bovine pancreatic ribonuclease (RNase A) (pdb entries 5nj1 and 5nj7). Circles, squares, and diamonds indicate His, Met, and Cys protein side chains, respectively. AP-1 bound amino acids are indicated with arrows.

To better assess the targetability of specific protein systems by AP-1, the regioselectivity based on the nucleophilic substitution must be paralleled by a study on the solvent exposure of the considered protein residues. Hence, solvent-accessible surface (SAS) analyses (Figure 6) were performed on the X-ray structures of the hen egg-white lysozyme (HEWL) and the bovine pancreatic ribonuclease (RNase A) proteins (pdb ids 5nj1 and 5nj7(16)), showing that the His residues that form adducts with AP-1 are closer to the surface. Indeed, the residues His12 and His48 in the bovine pancreatic ribonuclease are not metallated and, after a thorough investigation of the X-ray structure, we might conclude that the only reason is solvent inaccessibility. Cysteines and methionines are also situated in regions with less solvent accessibility, although not very deep, and are also not metallated.

## 3 Materials and Methods

### Materials

Lyophilized human carbonic anhydrase (hCA I), glyceraldehyde 3-phosphate dehydrogenase (GAPDH) from rabbit muscle, bovine superoxide dismutase (SOD), myoglobin (Mb) from horse heart, and human serum albumin (HSA) were acquired from Merck and utilized without additional purification or manipulation. AP-1 was synthesized in the MetMed laboratories at the Department of Chemistry, University of Florence in accordance with already established procedures.^13,14^

Dimethyl sulfoxide (DMSO) was acquired from Fluka. Liquid chromatography-mass spectrometry (LC-MS) materials (water and ammonium acetate) were procured from Honeywell.

### ESI MS Experimental Conditions

#### Sample Preparation

Stock solutions of hCA I 10^–4^ M, HSA 10^–3^ M, GAPDH 10^–4^ M, SOD 5 × 10^–3^ M, and Mb were prepared by dissolving the proteins and the peptide in H_2_O LC-MS grade. Stock solutions 10^–2^ M of the AP-1 compound was obtained dissolving the samples in DMSO.

For the experiments with hCA I, solutions of the protein 10^–5^ M and AP-1 at protein-to-metal ratio 1:3 were prepared and diluted with ammonium acetate solution 2 × 10^–3^ M (pH 6.8). The mixtures were then incubated at 37 °C up to 24 h.

For the experiments with GADPH, aliquots of the stock solutions were mixed with aliquots of AP-1 at protein-to-metal ratio 1:3 and diluted with ammonium acetate solution 2 × 10^–3^ M (pH 6.8) to 10^–5^ M final protein concentration. The mixtures were incubated at 37 °C up to 1 h.

For the experiments with Mb and SOD, solutions of the protein 10^–5^ M and AP-1 at protein-to-metal ratio 1:3 were prepared by diluting with ammonium acetate solution 2 × 10^–3^ M (pH 6.8). The mixtures were then incubated at 37 °C up to 24 h.

For the experiments with HSA, solution of the protein 10^–4^ M and AP-1 at the protein-to-metal ratio of 1:0.9 or 1:3 was prepared and diluted with ammonium acetate solution 2 × 10^–3^ M (pH 6.8). The mixture was then incubated at 37 °C up to 24 h.

##### ESI MS Analysis: Final Dilutions

After the incubation time, all solutions were sampled and diluted to a final protein concentration of 5 × 10^–7^ M for hCA I, HSA, GAPDH, and 10^–7^ M for Mb and SOD using ammonium acetate solution 2 × 10^–3^ M (pH 6.8).

In the final Mb, GAPDH and HSA solutions were also added with 0.1% v/v of formic acid shortly prior to the infusion in the mass spectrometer.

##### Instrumental Parameters

The ESI mass study was carried out utilizing a TripleTOF 5600^+^ high-resolution mass spectrometer (Sciex, Framingham, MA) furnished with a DuoSpray interface operating with an ESI probe. Respective ESI mass spectra were obtained via direct infusion at a flow rate of 5 μL/min.

The general ESI source parameters optimized for each protein and peptide analysis were as follows.

SOD parameters are positive polarity, ion spray voltage floating 5500 V, temperature 0, ion source gas 1 (GS1) 25 L/min; ion source gas 2 (GS2) 0, curtain gas (CUR) 15 L/min, collision energy (CE) 10 V, declustering potential (DP) 200 V, and range 1300–3400 *m*/*z*.

Mb parameters are positive polarity, ion spray voltage floating 5500 V, temperature 0, ion source gas 1 (GS1) 40 L/min, ion source gas 2 (GS2) 0, curtain gas (CUR) 15 L/min, collision energy (CE) 10 V, declustering potential (DP) 100 V, and range 700–2200 *m*/*z*.

GAPDH parameters are positive polarity, ion spray voltage floating 5500 V, temperature 0, ion source gas 1 (GS1) 20 L/min, ion source gas 2 (GS2) 0, curtain gas (CUR) 15 L/min, collision energy (CE) 10 V, declustering potential (DP) 100 V, and acquisition range 600–2000 *m*/*z*.

hCA I parameters are positive polarity, ion spray voltage floating 5500 V, temperature 0, ion source gas 1 (GS1) 25 L/min, ion source gas 2 (GS2) 0, curtain gas (CUR) 20 L/min, collision energy (CE) 10 V, declustering potential (DP) 300 V, and range 1500–3500 *m*/*z*.

HSA parameters are positive polarity, ion spray voltage floating 5500 V, temperature 0, ion source gas 1 (GS1) 40 L/min, ion source gas 2 (GS2) 0, curtain gas (CUR) 20 L/min, collision energy (CE) 10 V, declustering potential (DP) 200 V, and range 900–2600 *m*/*z*.

For acquisition, Analyst TF software 1.7.1 (Sciex) was employed, and deconvoluted spectra were attained by utilizing the Bio Tool Kit microapplication v.2.2 embedded in PeakView software v.2.2 (Sciex).

### Computational Methods

All computations were carried out with the Gaussian 09 A.02^34^ quantum chemistry package. Optimizations and the determination of electronic and solvation energies were performed in solvated phase (C-PCM)^35,36^ and by employing the density functionals as described below.

All geometrical optimizations were performed with the LANL2DZ effective core potential for Pt atom^37^ and the 6-31+G* basis set for other elements,^38,39^ while single-point electronic and solvation energy computations were performed with the LANL08(f) effective core potential for platinum^37,40^ and the 6-311++G** basis set for other elements.^41−43^ We used the range-corrected DFT functional CAM-B3LYP^44^ for geometrical optimization and electronic and solvation energies calculations. As we have shown elsewhere,^21^ the CAM-B3LYP/LANL08(f)/6-311++G**//CAM-B3LYP/LANL2DZ/6-31+G* functional-basis set combination yields the best results for geometry and energy computations for the aquation of arsenoplatin-1. DFT functionals are recognized to produce adequate geometries and reaction profiles for transition-metal-containing compounds^45−48^ including Pt-based anticancer compounds.^49−51^

Frequency calculations were carried out to confirm the convergence to the stationary points and to evaluate zero-point energy (ZPE) and thermal corrections to thermodynamic properties. Intrinsic reaction coordinate (IRC) computations were utilized to determine reactants and products minima connected with the transition states for each examined reaction step.

Single-point electronic energy computations were performed on the geometries optimized in the solution. The C-PCM continuum solvent methodology was employed to account for solvation.^35^ It was demonstrated to yield significantly smaller discrepancies than other continuum models for aqueous free energies of solvation for cations, anions, and neutrals and to be especially efficacious for the calculations of solution properties necessitating an enhanced accuracy of solution free energies.^52^ Free energies of solvation, considered as the difference between the solution energies and the gas phase energies, were added to the gas phase enthalpies and free energies values to have the corresponding values in the aqueous solution.

The solvent-accessible surface (SAS) of each examined amino acid was calculated by employing the SAS option available in Gromacs software.^53^

## 4 Conclusions

This study includes a combined experimental and theoretical investigation of arsenoplatin-1 interactions with protein targets. The analysis of the biomolecular interactions of AP-1 grounded on ESI MS measurements was extended here to a larger number of proteins than in the past, including carbonic anhydrase, superoxide dismutase, myoglobin, glyceraldehyde 3-phosphate dehydrogenase, and human serum albumin. The ESI MS results reveal that AP-1 generates in most cases tight adducts with the studied proteins containing the [(AP-1)-Cl]^+^ fragment, in nice agreement with previous observations made on HEWL and RNase A, and with the computational analysis carried out here. More in detail, the computational studies have considered the reactions of AP-1 with various nucleophiles, which mimic the side chains of neutral histidine, methionine, cysteine, and selenocysteine in neutral form as well as cysteine and selenocysteine in anionic form. The aquation of AP-1 is energetically disfavored with the reaction free energy of 19.2 kcal/mol, thus indicating that AP-1 presumably attacks its biomolecular targets by the direct substitution of the chloride ligand. The theoretical examination of thermodynamics and kinetics for the ligand substitution processes of AP with His, Met, Cys, Sec, Cys^–^, and Sec^–^ side chain models revealed that only neutral histidine and deprotonated cysteine and selenocysteine can effectively replace the chloride ligand in AP-1.

Moreover, a different and innovative result has been achieved here through the ESI MS experiments when reacting AP-1 with GAPDH and Mb. Indeed, in these latter cases, the adducts just contained a smaller fragment where the [As(OH)_2_] moiety is lost. This result is of particular interest as it provides direct evidence that arsenoplatin-1 may undergo degradation in the biological milieu, with the cleavage of the As–Pt bond giving rise to a protein-bonded platinum-containing fragment while releasing an arsenic-containing fragment.

Although the mechanistic details of the [As(OH)_2_] detachment from AP-1 were not expressly addressed in the present study, our calculations showed that the Pt–As distance is not significantly affected when replacing chloride by a nucleophilic protein ligand. This computational outcome suggests that the [As(OH)_2_] release is probably subsequent to protein metalation and may be kinetically influenced by the protein environment surrounding the Pt(II) binding site.

## Supporting Information Available

S1: Deconvoluted mass spectrum of human serum albumin and incubated with AP-1. S2: Enthalpy and Gibbs free energy values for the reaction of AP-1 with the investigated protein residue models in solution (PDF)
