# Supplementary material for: Reactions of Arsenoplatin-1 with Protein Targets: A Combined Experimental and Theoretical Study
Source: Inorg Chem. 2022 Feb 9;61(7):3240–8. doi: 10.1021/acs.inorgchem.1c03732 (PMC8864615; doi:10.1021/acs.inorgchem.1c03732)
Supplement: Supplementary file 1 — ic1c03732_si_001.pdf [file ic1c03732_si_001.pdf]

Supplementary Material

The reactions of Arsenoplatin-1 with Protein Targets: A combined experimental and theoretical study

Iogann Tolbatov[1], Damiano Cirri[2], Matteo Tarchi[3], Tiziano Marzo[4], Cecilia Coletti[5], Alessandro Marrone[5], Luigi Messori[3], Nazzareno Re[5]*, Lara Massai[3].*

[1] Institut de Chimie Moleculaire de l'Université de Bourgogne (ICMUB), Université de Bourgogne Franche-Comté (UBFC), avenue Alain Savary 9, Dijon, France; tolbatov.i@gmail.com

[2] Department of Chemistry and Industrial Chemistry, University of Pisa, Via G. Moruzzi 13, 56124 Pisa, Italy. Affiliation: damiano.cirri@dcci.unipi.it

[3] Department of Chemistry, University of Florence, Via della Lastruccia 3-13, 50019 Sesto Fiorentino, Italy. Affiliation: matteo.tarchi@unifi.it, luigi.messori@unifi.it, lara.massai@unifi.it

[4] Department of Pharmacy, University of Pisa, Via Bonanno Pisano 6, 56126, Pisa, Italy. Affiliation: tiziano.marzo@unipi.it

[5] CISUP - Centre for Instrumentation Sharing (Centro per l'Integrazione della Strumentazione Scientifica), University of Pisa, Italy;

[6] University Consortium for Research in the Chemistry of Metal ions in Biological Systems (CIRCMSB), Via Celso Ulpiani 27, 70126 Bari, Italy.

[7] Dipartimento di Farmacia, Università "G d'Annunzio" di Chieti-Pescara, via dei Vestini 31, Chieti, Italy; Affiliation: ccoletti@unich.it, amarrone@unich.it, nre@unich.it

* Correspondence: NR, nre@unich.it; LM, luigi.messori@unifi.it

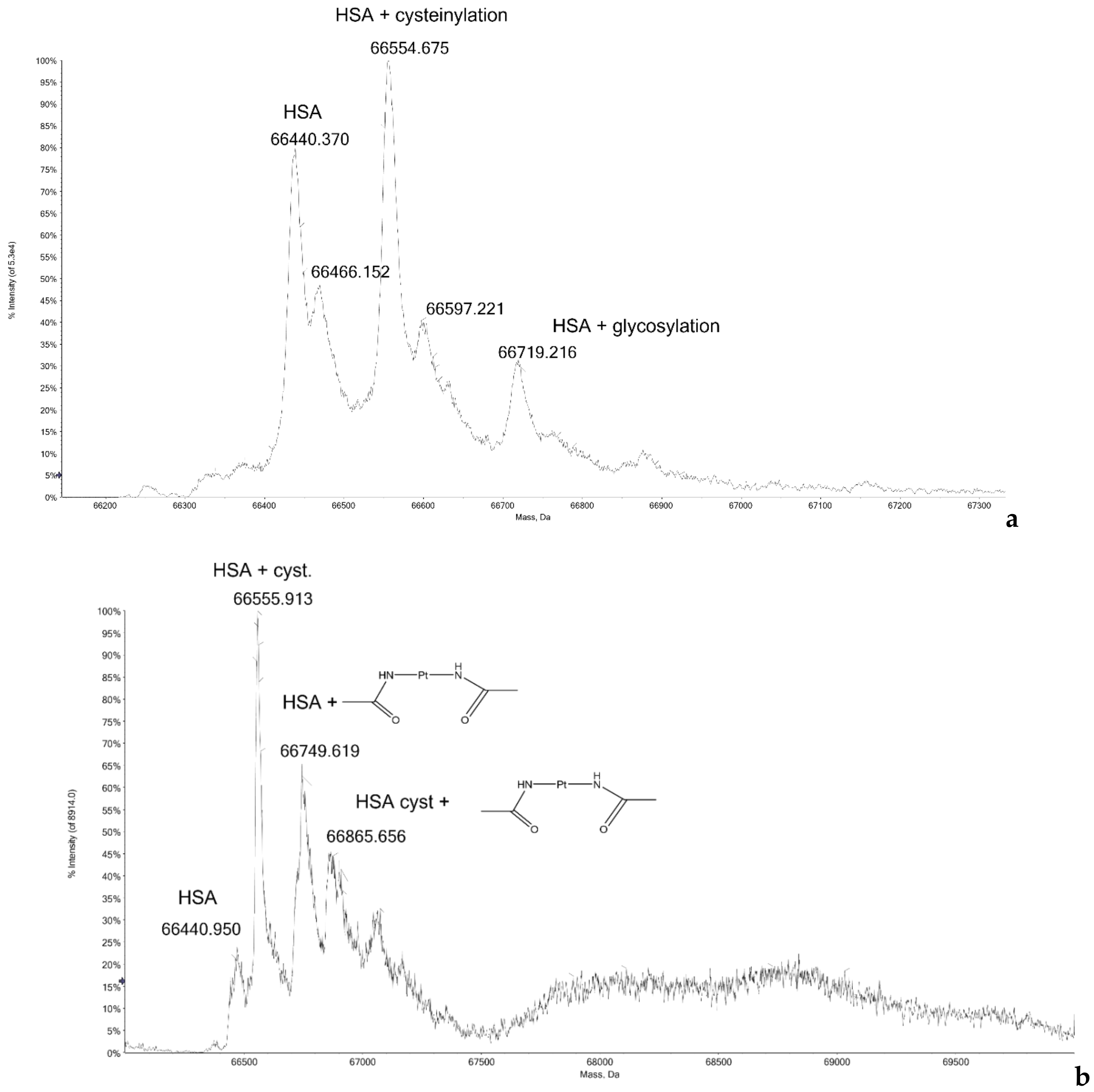

Figure S1 (a) Deconvoluted mass spectrum of human Serum Albumin, (b) deconvoluted mass spectrum of AP-1 incubated with HSA, at 37°C for 3h in 1:3 protein-to-AP-1 ratio

Table S1. Enthalpy and Gibbs free energy values for the reaction of AP-1 with the investigated protein residue models in solution. R, P, TS, RA, and PA stand for reactant, product, transition state, reactant-adduct, and product-adduct, respectively, in this table and hereafter. All values are reported in kcal/mol.

Reaction with	R->P		R->TS		RA->TS		RA->PA	
	ΔH	ΔG	ΔH	ΔG	ΔH	ΔG	ΔH	ΔG
4-His	-4.3	-0.8	7.7	18.9	7.0	11.4	-7.5	-3.4
5-His	-4.3	-0.7	6.4	17.4	7.1	11.6	-7.8	-4.1
Met	5.0	8.6	8.7	20.6	9.2	11.4	-1.2	0.0
Cys	8.0	11.3	11.7	22.6	11.5	14.1	1.6	3.1
Sec	6.6	10.0	10.5	21.6	10.8	13.4	-1.3	0.8
Cys^-	-19.2	-16.2	5.1	15.1	13.5	13.4	-16.1	-16.9
Sec^-	-18.3	-15.5	4.6	15.4	12.1	12.8	-16.3	-17.5
